# Efficacy of Single-Agent Chemotherapy in Endocrine Therapy-Refractory Metastatic Invasive Lobular Carcinoma

**DOI:** 10.1093/oncolo/oyad317

**Published:** 2023-12-09

**Authors:** Jason A Mouabbi, Wei Qaio, Yu Shen, Akshara Singareeka Raghavendra, Debasish Tripathy, Rachel M Layman

**Affiliations:** Department of Breast Medical Oncology, The University of Texas MD Anderson Cancer Center, Houston, TX, USA; Department of Biostatistics, The University of Texas MD Anderson Cancer Center, Houston, TX, USA; Department of Biostatistics, The University of Texas MD Anderson Cancer Center, Houston, TX, USA; Department of Breast Medical Oncology, The University of Texas MD Anderson Cancer Center, Houston, TX, USA; Department of Breast Medical Oncology, The University of Texas MD Anderson Cancer Center, Houston, TX, USA; Department of Breast Medical Oncology, The University of Texas MD Anderson Cancer Center, Houston, TX, USA

**Keywords:** invasive lobular carcinoma, ILC, breast cancer, hormone receptor positive, capecitabine, taxane

## Abstract

**Background:**

Hormone receptor (HR)-positive, HER2-negative metastatic invasive lobular breast cancer (mILC) is distinct from invasive ductal cancer (IDC) in clinicopathologic and molecular characteristics, impacting its response to systemic therapy. While endocrine therapy (ET) combined with targeted therapies has shown efficacy in ET-sensitive mILC, data on chemotherapy in ET-refractory mILC remain limited. We investigated the efficacy of single-agent capecitabine (CAP) versus taxanes (TAX) in ET-refractory HR+ HER2-negative patients with mILC.

**Materials and Methods:**

Using data from the MD Anderson prospectively collected breast cancer database, we identified patients with HR+ HER2-negative mILC who received prior ET and first-time chemotherapy in the metastatic setting. We compared outcomes between 173 CAP-treated and 96 TAX-treated patients.

**Results:**

CAP-treated patients had significantly better median progression-free survival (PFS) than TAX-treated patients (8.8 vs 5.0 months, HR 0.63, *P* < .001). Overall survival (OS) did not differ significantly between the groups (42.7 vs 36.6 months for CAP vs TAX, respectively, HR 0.84, *P* = .241). Multivariate analyses for PFS and OS revealed better outcomes in subjects with fewer metastatic sites and those exposed to more lines of ET. Additionally, Black patients showed worse OS outcomes compared to White patients (HR 2.46; *P* = .001).

**Conclusion:**

In ET-refractory HR+ HER2-negative mILC, single-agent CAP demonstrated superior PFS compared to TAX. Our findings highlight the potential benefit of CAP in this patient subset, warranting further investigation through prospective trials.

## Teaser

This study sheds light on the treatment of hormone receptor-positive, HER2-negative metastatic invasive lobular breast cancer for patients who no longer respond to endocrine therapy.

**Implications for Practice**

This study sheds light on the treatment of hormone receptor-positive, HER2-negative metastatic invasive lobular breast cancer (mILC) for patients who no longer respond to endocrine therapy. The findings reveal that single-agent capecitabine may offer a promising option, significantly improving progression-free survival compared to taxanes. These results have meaningful clinical implications, suggesting that capecitabine could be a viable treatment for ET-refractory mILC. Importantly, our study emphasizes the need for personalized approaches, considering racial disparities in treatment response. By guiding clinicians in selecting appropriate therapies, this research may ultimately improve outcomes and quality of life for patients with mILC.

## Introduction

Invasive metastatic breast cancer (mBC) is composed of multiple histological subtypes. The most common is invasive ductal carcinoma (IDC), also commonly classified as invasive carcinoma of no special type, which accounts for 80% of all invasive BCs,^1^ followed by invasive lobular carcinoma (ILC) which accounts for approximately 10%.^2^ ILC is distinct from IDC in its clinicopathologic characteristics and molecular alterations.^3,4^ One special feature of ILC is the near-universal loss of the cell adhesion protein E-cadherin in approximately 90% of cases^5^ because of a loss of function via genomic loss (most commonly heterogenous 16q [90%-94% of cases]^5-8^) or mutation.^3^ ILC generally has features that are associated with a good prognosis, most often with low grade, low proliferation index, and strong ER positivity.^9^ However, compared to IDC, ILC tends to have a higher risk of distant recurrence after 10 years^10^ and tends to exhibit peculiar metastatic patterns.^11^

The majority (93%) of metastatic ILC (mILC) is hormone receptor-positive and human epidermal growth factor receptor 2-negative (HR+/HER2−).^10^ As long as mILC is considered hormone sensitive, they are treated with sequential lines of endocrine therapy (ET) in combination with targeted therapies (TT) such as cyclin-dependent kinase 4/6 inhibitors (CDK4/6is), the mammalian target of rapamycin inhibitor (mTORi) everolimus, and the phosphoinositide 3-kinase inhibitor alpelisib.^12^

Once these tumors become ET-refractory, they are treated with sequential single-agent chemotherapies.^1^ However, there is no consensus on the optimal first-line (1L) chemotherapy regimen for HR+/HER2− mBC. Two commonly used agents with favorable toxicity profiles are taxanes (TAX) and capecitabine (CAP). Most studies assessing the effectiveness of chemotherapy in HR+/HER2− mBC did not report outcomes based on histology.^13-17^ However, it is important to examine the effectiveness of different chemotherapeutic agents in mILC specifically given the compelling evidence that early stage ILC treated with chemotherapy responded more poorly compared IDC.^18^

In this study, we compare the outcomes of hormone refractory HR+/HER2− mILC patients treated with 1L taxanes and compare it to those who received 1L CAP.

## Materials and Methods

### Study Population and Variables

We searched the IRB-approved prospectively collected breast cancer database (MDA IRB# PA17-0199) at The University of Texas MD Anderson Cancer Center (Houston, TX) to identify patients with HR+/HER2− mILC that were ET-refractory and receiving 1L chemotherapy between January 1997 and June 2020. Data including patient demographics, treatment received (CAP vs taxane), metastatic presentation (de novo vs recurrent), number of metastatic sites (1, 2, or 3 and more), location of metastatic sites (non-visceral vs visceral), number of prior hormonal therapies, exposure to prior CDK4/6i, survival, and last follow-up were collected. Patients were divided into 2 groups: those who received capecitabine (CAP group) and those who received a taxane (TAX group). The TAX group included patients who received paclitaxel, nab-paclitaxel, or docetaxel.

### Statistical Analysis

The distribution of each continuous variable was summarized by its median and range. The distribution of each categorical variable was summarized in terms of its frequencies and percentages. Continuous variables were compared between groups using Wilcoxon rank sum test, and Fisher’s exact test was used to assess the association between categorical variables. The Kaplan-Meier product-limit method was used to estimate the distributions of progression-free survival (PFS) and overall survival (OS) distributions, and the log-rank test was used to compare the distributions by the two treatment groups. The Cox proportional hazards regression model was used to evaluate the association of patient prognostic variables and OS or PFS. PFS was defined as the time from the date of initiation of the correspondent systemic therapy to the time of disease progression or death or treatment ending in the current line therapy for any reason. OS is defined as the time from date of distant metastasis till death or censored at the last follow-up. The variables indicating potentially significant association with the outcome (ie, *P*-value ≤ .05) were included in the initial saturated multivariable model while the chemotherapy effect was forced in the model since it is the research interest for the study. The backward model selection was then employed to identify the final multivariable model wherethe remaining variables have *P* < .05. All computations were carried out in SAS version 9.4.

## Results

### Baseline Characteristics

We reviewed 269 subjects, of whom 173 received CAP and 96 received TAX as 1L chemotherapy. In the overall population, the median age was 52, with a range from 27 to 81 years. Eighty percent of patients were White, 6% Black, 9% Hispanic, and 3% Asian. Eighty-two percent of patients had recurrent breast cancer and 18% were de novo metastatic. Approximately 50% of patients had 3 or more metastatic lesions. Sixty percent of patients had non-visceral metastasis and 40% had visceral ones. Approximately 50% of patients received only one prior ET and 22% had exposure to prior CDK4/6is (Table 1). When comparing the patients’ characteristics between the CAP group and the TAX group, the only statistically significant different variable was seen in the metastatic origin; 80% of patients in the CAP group (20% de novo) versus 70% of patients in the TAX group had recurrent disease (*P* = .001; Table 2).

**Table 1.** Patient characteristics.

	Metastatic ET-refractory ILC treated with 1L chemotherapy *N* = 269
Age—median (min, max)	52 (27, 81)
Race—no (%)	
White	215 (80)
Black	17 (6)
Hispanic	23 (9)
Asian	9 (3)
Other	5 (2)
Metastatic presentation	
De novo	50 (18)
Recurrent	219 (82)
Number of metastases	
1	60 (22)
2	78 (29)
3 or more	131 (49)
Location of metastatic site	
Non-visceral	165 (60)
Visceral	104 (40)
Number of prior endocrine therapies	
1	129 (48)
2	84 (31)
3 or more	56 (21)
Exposure to prior CDK4/6i	
No	211 (78)
Yes	58 (22)
Chemotherapy agent	
Capecitabine	173 (64)
Taxane	96 (36)

Abbreviations: 1L, first line; CDK4/6i, cyclin-dependent kinase 4 and 6 inhibitor; ET, endocrine therapy; ILC, invasive lobular carcinoma.

**Table 2.** Patient characteristics per chemotherapy agent.

	Capecitabine *N* = 173	Taxane *N* = 96	*P*-value
Age—median (min, max)	52 (27, 81)	52 (32, 77)	.801
Race			
White	140 (81)	75 (78)	.329
Black	10 (6)	7 (7)	
Hispanic	13 (7)	10 (11)	
Asian	8 (5)	1 (1)	
Other	2 (1)	3 (3)	
Metastatic presentation			
De novo	22(10)	28 (30)	.001*
Recurrent	151 (90)	68 (70)	
Disease-free interval			
0-12 months	8 (5.3)	3 (4.4)	.274
12-24 months	16 (10.6)	5 (7.4)	
24-60 months	52 (34.4)	33 (48.5)	
>60 months	75 (49.7)	27 (39.7)	
Number of metastases			
1	44 (25)	16 (16)	.204
2	46 (26)	32 (33)	
3 or more	83 (49)	48 (51)	
Location of metastatic site			
Non-visceral	111 (64)	54 (56)	.239
Visceral	62 (36)	42 (44)	
Number of prior endocrine therapies			
1	82 (49)	47 (50)	.398
2	50 (28)	34 (35)	
3	30 (17)	10 (10)	
4	11 (6)	5 (5)	
Exposure to prior CDK4/6i			
No	130 (75)	81 (85)	.089
Yes	43 (25)	15 (15)	
Years patients were treated			
≤2002	17 (9.8)	11 (11.5)	.109
2003-2008	42 (24.3)	30 (31.3)	
2009-2014	74 (42.8)	44 (45.8)	
≥2015	40 (23.1)	11 (11.5%)	
Prior exposure to taxanes in early stage			
Yes	91 (52)	25 (26)	<.001*
No	82 (48)	71 (74)	

Abbreviation: CDK4/6i, cyclin-dependent kinase 4 and 6 inhibitor. Italic/starred values indicates statistical significance

### Outcomes

**Figure 1.**
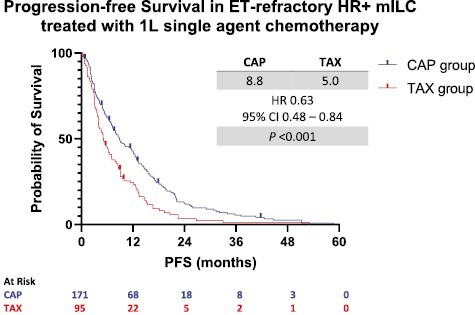
Progression-free Survival in patients receiving capecitabine versus taxane. Abbreviations: ET, endocrine therapy; HR+, hormone receptor positive; mILC, metastatic invasive lobular carcinoma; CAP, capecitabine; TAX, taxanes; PFS, progression-free survival; HR, Hazard ratio; 95% CI, 95% CI

After a median follow-up of 103.7 months (95% CI 81.9-121.8), there were 158 progression events in the CAP group and 92 events in the TAX group. Patients who received 1L CAP had better median PFS compared to those who received TAX (8.8 vs 5.0 months, HR 0.63, 95% CI 0.48-0.82, *P* < .001 for the log-rank test) (Fig. 1). In the univariate analysis, the mono-chemotherapeutic agent, number of metastatic sites and number of prior endocrine therapies were correlated with PFS (Table 3). According to the multivariate analysis, the use of CAP was an independent favorable prognostic factor for PFS compared to the use of TAX (HR 0.70, 95% CI 0.53-0.91, *P* = .008) after adjusting for other risk factors. Similarly, a higher number of prior endocrine therapies was associated with better PFS. Conversely, a higher number of metastatic sites was associated with worse PFS (Table 4).

**Table 3.** Univariate Cox for PFS and OS.

	PFS			OS		
	Hazard ratio (95% CI)	*P*-value	Overall *P*-value	Hazard ratio (95% CI)	*P*-value	Overall *P*-value
Chemotherapeutic agent						
Capecitabine vs taxane	0.63 (0.48-0.82)		<.001*	0.85 (0.64-1.12)		.241
Age	0.99 (0.98-1.00)		.161	1.02 (1-1.03)		.020*
Race						
Black vs White	2.04 (1.22-3.41)	.006	.074	3.05 (1.82-5.13)	<.001	<.001*
Hispanic vs White	1.08 (0.69-1.69)	.745		0.57 (0.35-0.96)	.033	
Asian vs White	1.44 (0.71-2.94)	.310		0.91 (0.37-2.23)	.841	
Other vs White	0.78 (0.29-2.11)	.627		0.60 (0.08-4.26)	.605	
Metastatic presentation						
De Novo vs Recurrent	1.08 (0.79-1.49)		.620	0.78 (0.56-1.10)		.159
Number of metastases						
1 vs 3 or more	0.62 (0.45-0.86)	.004	.007*	0.79 (0.55-1.14)	.207	.365
2 vs 3 or more	0.72 (0.54-0.97)	.003		0.85 (0.63-1.17)	.323	
Location of metastatic site						
Non-Visceral vs Visceral	0.83 (0.64-1.07)		.152	0.71 (0.54-0.94)		.015*
Number of prior endocrine therapies						
1 vs 4	2.59 (1.39-4.82)	.003	.004*	5.93 (2.74-12.81)	<.001	<.001*
2 vs 4	2.97 (1.57-5.63)	.001		4.1 (1.87-8.96)	.004	
3 vs 4	1.96 (1.00-3.86)	.051		2.32 (1.02-5.25)	.044	
Exposure to prior CDK4/6i						
No vs yes	1.1 (0.79-1.54)		.577	0.95 (0.65-1.39)		.799
Prior exposure to taxanes in early stage						
No vs yes	1.25 (0.97-1.60)		.087	1.01 (0.77-1.33)		.946

Abbreviation: CDK4/6i, cyclin-dependent kinase 4 and 6 inhibitor. Italic/starred values indicates statistical significance

**Table 4.** Multivariable Cox model for PFS.

Parameter	Hazard ratio (95% CI)	*P*-value
Chemotherapeutic agent		
Capecitabine vs taxane	0.69 (0.53-0.91)	.008*
Number of metastases		
1 vs 3 or more	0.64 (0.45-0.89)	.008*
2 vs 3 or more	0.68 (0.51-0.91)	.010*
Number of prior endocrine therapies		
1 vs 4	2.54 (1.36-4.74)	.003*
2 vs 4	2.95 (1.54-5.62)	.001*
3 vs 4	2.00 (1.01-3.96)	.046*

**Figure 2.**
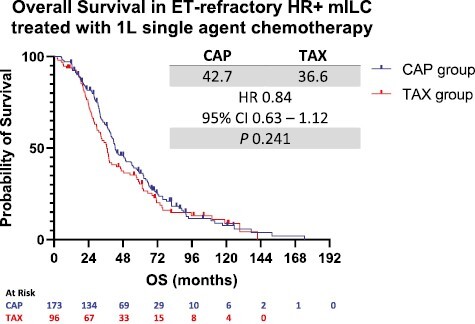
Overall Survival in patients receiving capecitabine versus taxane. Abbreviations: ET, endocrine therapy; HR+, hormone receptor positive; mILC, metastatic invasive lobular carcinoma; CAP, capecitabine; TAX, taxanes; OS, overall survival; HR, hazard ratio; 95% CI, 95% CI

In terms of OS, after a median follow-up of 103.7 months (95% CI 81.9-121.8) months, there were 136 deaths in the CAP group and 80 in the TAX group. There was no statistically significant OS difference in patients who received CAP versus those who received TAX (42.7 vs 36.6 months, HR 0.85, 95% CI 0.64-1.12, *P* = .214 for the log-rank test) (Fig. 2). In the univariate analysis, age, race, location of metastatic sites, and number of prior endocrine therapies were correlated with OS (Table 3). According to the multivariate analysis, the use of CAP versus TAX was not significantly associated with OS (HR 0.84, 95% CI 0.63-1.14, *P* = .272). Black race was an independent poor prognostic factor for OS compared to White race (HR 2.46, 95% CI 1.40-4.32, *P* = .001). Similar to PFS, a higher number of prior endocrine therapies was associated with better OS (Table 5).

**Table 5.** Multivariable Cox model for OS.

Parameter	Hazard ratio (95% CI)	*P*-value
Chemotherapeutic agent		
Capecitabine vs taxane	0.84 (0.63-1.14)	*.272*
*Race*		
Black vs White	2.46 (1.40-4.32)	.001*
Hispanic vs White	0.64 (0.37-1.10)	*.108*
Asian vs White	0.72 (0.28-1.85)	*.506*
Other vs White	0.63 (0.08-4.59)	*.653*
Number of prior endocrine therapies		
1 vs 4	6.09 (2.8-13.22)	<.001*
2 vs 4	3.58 (1.62-7.92)	.001*
3 vs 4	2.25 (0.99-5.12)	.052
Location of metastatic site		
Visceral vs non-visceral	1.28 (0.96-1.71)	.086

## Discussion

This is the largest retrospective analysis to date to report outcomes of ET-refractory HR+ HER2− mILC subjects treated with the single-agent chemotherapies. Our analysis suggests that mILC subjects treated with CAP had better median PFS but not OS compared to those treated with TAX. We observed that the difference in PFS between CAP and TAX treatment is consistent with the results published by the Cochrane Breast Cancer Group that showed CAP to have a slight PFS advantage over TAX in HR+ mBC.^19^ The lack of difference in OS between CAP and taxane treatment is also consistent with results from other studies.^15,20^

To date, there is only one other publication that assessed the outcomes of patients with mILC to single-agent chemotherapy.^21^ In this study, the authors analyzed 118 patients with mILC from 3 clinical trials and they reported a median PFS of 4.1 months and median OS of 13.4 months when treated with single-agent eribulin.^21^ The difference in OS reported in the aforementioned study compared to our study accounts for the fact that all the patients who received eribulin were exposed to a least one prior chemotherapeutic agent, whereas in our study all patients were chemotherapy naïve in the metastatic setting.

The reasons for the difference in PFS between CAP and taxane treatment are not well understood. Capecitabine is an oral prodrug that is converted to its active form 5-fluorouracil in tumor tissues, mainly through the enzyme thymidine phosphorylase (TP). Studies have shown that TP expression in breast cancer cells may represent a biomarker of sensitivity to CAP treatment.^22,23^ Thus, one possible hypothesis is that ILC cells contain higher levels of TP, making CAP more effective in delaying progression. Another possibility is that CAP might be more effective at targeting the molecular pathways that drive the growth of mILC. Other treatment selection biases are possible when analyzing a retrospective patient cohort.

Recently, the antibody-drug conjugates trastuzumab deruxtecan (T-DXd) and sacituzumab govitecan have been shown to outperform chemotherapy in HR+ HER2− mBC. Our study is still relevant given that the ADCs were only studied after one prior chemotherapeutic agent in the metastatic setting.^24,25^

The finding on multivariate analysis that when compared to White race, Black race was an independent poor prognostic factor for OS in ET-refractory patients with mILC is concerning and raises important questions about possible race-associated biological differences.^26,27^ This finding reinforces the importance of conducting studies that aim to understand the underlying biological and genetic factors that may contribute to differences in cancer behavior among different racial and ethnic groups to tailor personalized treatment strategies.

We acknowledge several limitations to our study. The retrospective design of our study limits our ability to draw definitive conclusions about the superiority of one chemotherapeutic agent over the other. Moreover, the patients included in our study were based on single institution, hence the population might not be representative of all of patients with mILC. Finally, the medical records of some patients were incomplete, which limited the sample size.

## Conclusion

The results of this study suggest that CAP may be a preferred treatment option for patients with mILC based on the longer PFS observed. However, it is important to note that further research or/and prospective clinical trial is needed to confirm the findings of this study.

## Data Availability

The data that support the findings of this study are available from the corresponding author, upon reasonable request.
